# Evidence-Based Recommendations for the Pharmacological Treatment of Women with Schizophrenia Spectrum Disorders

**DOI:** 10.1007/s11920-023-01460-6

**Published:** 2023-10-21

**Authors:** Bodyl A. Brand, Elske J. M. Willemse, Iris M. H. Hamers, Iris E. Sommer

**Affiliations:** Department of Biomedical Sciences and Systems, Cognitive Neurosciences, University of Groningen, University Medical Center Groningen (UMCG), Neuro Imaging Center 3111, Deusinglaan 2, 9713 AW Groningen, the Netherlands

**Keywords:** Antipsychotic medication, Schizophrenia, Sex differences, Estrogen, Raloxifene, Prolactin

## Abstract

**Purpose of Review:**

Despite clear evidence that sex differences largely impact the efficacy and tolerability of antipsychotic medication, current treatment guidelines for schizophrenia spectrum disorders (SSD) do not differentiate between men and women. This review summarizes the available evidence on strategies that may improve pharmacotherapy for women and provides evidence-based recommendations to optimize treatment for women with schizophrenia.

**Recent Findings:**

We systematically searched PubMed and Embase for peer-reviewed studies on three topics: (1) sex differences in dose-adjusted antipsychotic serum concentrations, (2) hormonal augmentation therapy with estrogen and estrogen-like compounds to improve symptom severity, and (3) strategies to reduce antipsychotic-induced hyperprolactinemia. Based on three database studies and one RCT, we found higher dose-adjusted concentrations in women compared to men for most antipsychotics. For quetiapine, higher concentrations were specifically found in older women. Based on two recent meta-analyses, both estrogen and raloxifene improved overall symptomatology. Most consistent findings were found for raloxifene augmentation in postmenopausal women. No studies evaluated the effects of estrogenic contraceptives on symptoms. Based on two meta-analyses and one RCT, adjunctive aripiprazole was the best-studied and safest strategy for lowering antipsychotic-induced hyperprolactinemia.

**Summary:**

Evidence-based recommendations for female-specific pharmacotherapy for SSD consist of (1) female-specific dosing for antipsychotics (guided by therapeutic drug monitoring), (2) hormonal replacement with raloxifene in postmenopausal women, and (3) aripiprazole addition as best evidenced option in case of antipsychotic-induced hyperprolactinemia. Combining these strategies could reduce side effects and improve outcome of women with SSD, which should be confirmed in future longitudinal RCTs.

**Supplementary Information:**

The online version contains supplementary material available at 10.1007/s11920-023-01460-6.

## Introduction

While some patients prefer to be treated without medication, most acute psychotic episodes are treated with antipsychotics. After remission of acute psychosis, many patients are prescribed antipsychotic medication for a longer period to prevent a new psychotic episode. Classic antipsychotics such as haloperidol are still being used, yet, second-generation antipsychotics such as olanzapine and amisulpride are prescribed with increasing frequency given their better efficacy–tolerability balance, particularly with respect to extrapyramidal symptoms [1]. Despite seminal work from pioneers like Dr. Seeman, Dr. Reicher-Rössler, and Dr. Kulkarni [2–7], current antipsychotic treatment guidelines (e.g., NICE [8] and PORT [9]) do not provide sex-specific recommendations. These guidelines are bound to studies performed in study populations that predominantly consist of male patients that usually lack well-powered sex-specific sub-analyses [10–14].

Currently, young and elderly patient groups with schizophrenia spectrum disorders (SSD) are addressed in specifically adjusted guideline recommendations [15, 16], yet tailored guidelines for women are still lacking. There is clear evidence that sex differences in body structure and hormones largely impact the efficacy and tolerability of antipsychotic medications, while benefits of estrogen suppletion could be important for women during and after menopause. Large biologically determined (i.e., sex) differences in body composition, sex hormones, and hormonal transitions affect the pharmacokinetics of antipsychotics; with differences in absorption, distribution, and metabolism [17]. The slower metabolism and lower renal clearance may cause higher blood levels for antipsychotics in women. Even when antipsychotic blood levels are equal, sex differences in pharmacodynamics, involving the blood–brain barrier, receptor sensitivity, and binding profile, may lead to higher sensitivity of the dopaminergic system in women [17, 18]. As current guidelines on dosing of antipsychotics do not take these sex differences into account [19, 20], women are at increased risk to be overdosed.

Another sex-specific aspect of psychosis treatment concerns the protective effects of estrogens on mental health in general and on positive, negative, and cognitive symptoms of SSD in particular [21]. In premenopausal women, symptoms fluctuate with menstrual cycle phases and tend to exacerbate during low estrogenic phases of the menstrual cycle [22•]. After menopause, the decline in estrogens leads to a deterioration in the clinical course with more hospitalizations and a higher need for sheltered housing [23, 24]. Some previous studies showed that estrogen augmentation therapy is effective in improving symptomatology in these women [25, 26]. However, augmentation with estrogens may not be safe for long-term use due to its carcinogenic effects on the sex organs. In addition, it does not prevent conception in premenopausal women. Combined contraceptives might be a safe and effective alternative for young women. Combined contraceptives contain synthetic formulations of estrogens (ethinyl estradiol, EE) and progesterone (progestin) can stabilize estrogen levels in women that experience strong hormonal fluctuations and could therefore improve symptomatology [27]. Another relatively safe alternative for estrogen augmentation is augmentation with the selective estrogen receptor modulator (SERM) raloxifene. Raloxifene has agonistic effects on the brain and bones, but exerts antagonistic effects on the breasts and uterus [28–30]. Together, these three options of estrogenic augmentation therapy may provide opportunities to improve treatment outcomes for women with SSD, especially after menopause.

Finally, many antipsychotics cause a sustained increase in prolactin secretion [14]. Hyperprolactinemia is more common in women, with prevalence rates ranging from 42 to 93% compared to 18 to 72% in men [31]. High prolactin levels inhibit estrogen production [23], which can lead to adverse sexual, somatic, and mental health effects such as menstrual cycle irregularities, sexual dysfunction, osteoporosis, menopausal complaints, depressed mood, and increased risks for cardiovascular diseases and breast cancer [32–35], For example, 48% of women receiving antipsychotic treatment report menstrual irregularities [36], and 32% of women treated with prolactin-raising antipsychotics for > 10 years have reduced bone mineral density [37]. Women who used prolactin-raising antipsychotics for more than 5 years have a 150% increased risk for breast cancer [35], which is especially worrisome since female SSD patients are already at increased genetic risk for this disease [38]. Lowering the dose of prolactin-enhancing agents increases relapse risk [39] and is not consistently demonstrated to be effective as prolactin levels can already be raised when using a low dose of certain antipsychotics [40]. Preventing or correcting antipsychotic-induced hyperprolactinemia is therefore an important goal in pharmacotherapy of women with SSD. Several strategies have been described, for example, switching to a prolactin-sparing antipsychotic or the addition of a component like aripiprazole or vitamin B6 [41•].

In sum, pharmacological treatment for women with SSD may benefit from a sex-sensitive perspective, which can only be constructed if we know how to account for female-specific treatment responses to antipsychotic treatment. This paper aims to provide evidence-based recommendations for pharmacotherapy for women with SSD. For that purpose, this systematic review summarizes findings on three main topics: (1) sex differences in dose-adjusted blood levels of antipsychotics, (2) the potential benefits of hormonal replacement therapy with estrogen, and (3) the estrogen-like compounds and reviews potential strategies to correct antipsychotic-induced prolactin raises.

## Methods

### Literature Search

A systematic search was conducted using PubMed (Medline) and Embase for three main topics:

1. Sex differences in dose-adjusted antipsychotic serum concentrations
2. Hormonal augmentation therapy with estrogen, raloxifene, or combined systemic contraceptives in women with SSD
3. Strategies to reduce antipsychotic-induced hyperprolactinemia in patients with SSD

Combinations of the following search terms were used: “serum concentration,” “dose concentration,” “serum level,” “antipsychotic,” and “therapeutic drug monitoring” (topic 1); “schizophrenia,” “psychosis,” “schizoaffective,” “schizophreniform,” “estrogen,” “raloxifene,” and “combined contraceptive” (topic 2); and “schizophrenia,” “psychosis,” “schizoaffective,” “schizophreniform,” “prolactin,” “switch,” “aripiprazole,” “dopamine agonist,” and “metformin” (topic 3). See supplementary information for the search strings in full detail. No year or language restrictions were applied. The search cutoff data for all topics was 30 May 2022; a search update was performed according to PRISMA guidelines [42].

### Inclusion Criteria

In general, studies were included if they met the following inclusion criteria: (1) studies were published in a peer-reviewed journal; (2) studies reported data from at least one independent sample; (3) the study included adult patients (≥ 18 years old) with a diagnosis of SSD (schizophrenia, schizoaffective disorder, schizophreniform disorder, or psychotic disorder NOS), according to the diagnostic criteria of the Diagnostic and Statistical Manual of Mental Disorders (DSM-III, DSM-III-R, DSM-IV, DSM-IV-TR, DSM-5) [43] or the International Classification of Diseases (ICD-9 or ICD-10) [44]. Additional inclusion criteria per topic can be found in the supplement. When high-quality meta-analyses were available and published in the last 5 years (between January 2018 and the search cut-off date), we used and updated these, instead of reviewing all individual studies.

## Results

### Dose-Adjusted Sex Differences in Antipsychotic Serum Concentrations

We retrieved three database studies [45•, 46, 47] and one randomized controlled trial (RCT) [48] providing sex-specific dose-adjusted antipsychotic serum concentrations (Fig. 2 in supplement, Table 1). Castberg and colleagues [46] included 11,968 patients (48% women) using clozapine, olanzapine, risperidone, or quetiapine from a routine therapeutic drug monitoring (TDM) database and found that dose-adjusted serum concentrations for clozapine, olanzapine, and risperidone were significantly higher in women compared to men (28.2%, 26.1%, and 18.7%, respectively, higher in women). For quetiapine, no significant sex differences were found in younger age groups (age < 40 years). Yet, age-by-sex interaction indicated that sex differences in dose-adjusted concentrations for quetiapine increase with age. At the age of 80, dose-adjusted concentrations for quetiapine are 33% higher in women compared to men.

A similar TDM study by Jönsson and colleagues [45•] included 26,833 patients (47% women) and reported significantly higher dose-adjusted concentrations in women for 10 out of 12 antipsychotics compared to men. The largest increases were found for olanzapine and clozapine, showing higher dose-adjusted concentrations of, respectively, 59% and 40% in women. For quetiapine, no significant differences were found, with dose-adjusted concentrations in women being 6.4% lower. For sertindole, the absence of a significant difference between men and women was possibly due to the small sample size included for this antipsychotic, as sex differences were numerically similar to the other evaluated antipsychotics.

An observational study by Tveito and colleagues [48] investigated long-acting injectable formulations of paliperidone (once-monthly) in 1158 patients (36% women, 3394 samples). They found significantly higher dose-adjusted concentrations in women (14% higher than in men), while men were prescribed 10% higher dosages than women. Dose-adjusted concentrations in older women (≥ 50 years) were increased by 29%, 30%, and 41%, compared to older men (≥ 50 years), younger women (≤ 49 years), and younger men (≤ 49 years), respectively (all *p*’s < 0.001).

An RCT by Hoekstra and colleagues [47] examined dose-adjusted concentrations in 144 patients (35.4% women), randomized to receive either amisulpride, aripiprazole, or olanzapine. The sample sizes of this study were much smaller as compared to the other studies, with 12 women (29%) in the olanzapine group, 12 women (24%) in the aripiprazole group, and 27 (51%) in the amisulpride group. No significant differences were found for olanzapine, while dose-adjusted serum levels were significantly higher in women for aripiprazole (56%) and amisulpride (72%). Hoekstra and colleagues also reported significantly higher prolactin levels in women than in men, especially for amisulpride, and a significantly higher BMI increase in women on this antipsychotic as compared to the two other antipsychotics.

**Table 1:** Studies investigating sex differences in serum concentrations of antipsychotic drugs

**Study**	***N***	**Age range (years)**	**Women (%)**	**Included antipsychotics and *n* of subgroups**	**Results for dose-adjusted concentrations**
Castberg et al*.* (2017) [46]	11,968	18–100	47.9	Clozapine (*n* = 32,416), olanzapine (*n* = 36,705), risperidone (*n* = 17,654), and quetiapine (*n* = 7628)	20–30% higher in women than men, 3.8% higher for quetiapine in younger women (age of 40) (NS), 33% higher for quetiapine in older women (age of 80)
Jönsson et al*.* (2019) [45•]	26,833	0–99	46.6	Amisulpride (*n* = 506), aripiprazole (*n* = 1610), clozapine (*n* = 1189), flupentixol (*n* = 215), haloperidol (*n* = 390), olanzapine (*n* = 10,268), perphenazine (*n* = 1065), quetiapine (*n* = 5853), risperidone (*n* = 3255), sertindole (*n* = 111), ziprasidone (*n* = 1235), and zuclopenthixol (*n* = 691)	Higher concentration/dose ratios in women for all antipsychotics. Largest differences for olanzapine (59%) and clozapine (40%). No differences found for sertindole (NS) and quetiapine (NS, 6% lower in women)
Tveito et al*.* (2021) [48]	1223	15–90	36.5	Paliperidone long acting (*n* = 1223)	14% higher in women compared to men 29%, 30%, and 41% in older women (≥ 50 years) compared to older men, younger women (≤ 49 years), and younger men (≤ 49 years)
Hoekstra et al*.* (2021) [47]	144	18–65.6	35.4	Amisulpride (*n* = 52), aripiprazole (*n* = 51), and olanzapine (*n* = 41)	For amisulpride 71.9% higher in women For aripiprazole 55.8% higher compared to men; 7.7% higher for olanzapine (NS)

*n* number in the sample, *NS* not significant

### Hormonal Augmentation Therapies

#### Estrogen Augmentation

No high-quality meta-analyses were performed on this topic in the last 5 years. We found seven RCTs examining the effects of estrogen augmentation therapy on symptom severity (Fig. 3 in supplement), which were all performed in premenopausal women or in women of premenopausal age (Table 2). Types of estrogen augmentation that were examined were augmentation with estradiol (E2), conjugated equine estrogens (CEE), ethinyl estradiol (EE), and estradiol valerate (EV). In most studies, estrogens showed to be superior compared to placebo in reducing total symptomatology and symptomatology subscales (i.e., positive, negative, and general symptoms) as measured by the Positive and Negative Syndrome Scale (PANSS) [49]. However, Louzã and colleagues [50] did not find a significant effect of CEE on symptom severity compared to placebo in a sample of 40 women (age range 18–49, menopause status unknown), although a trend towards improvement was visible. It is important to highlight that the most recent meta-analysis concerning estrogen augmentation dates back to 2014 [26]. There have not been any meta-analyses on this subject within the last 5 years, possibly due to the limited availability of recent RCTs published on this topic, as only one additional RCT has been published [63]. The meta-analysis performed by Heringa and colleagues [26] found positive effects of estrogen augmentation, with effect sizes ranging from moderate to high for total symptom severity (Hedges’ *g* = 0.83, *p* = 0.001), positive symptoms (Hedges’ *g* = 0.48, *p* = 0.001), and negative symptoms (Hedges’ *g* = 0.39, *p* = 0.011), based on a sample of 427 women with SSD. Overall, these studies show that estrogenic augmentation therapies reduced PANSS total, positive, negative, and general scores specifically in premenopausal women with SSD.

**Table 2:** Randomized controlled trials investigating the effects of estrogen augmentation therapy on symptoms in women with SSD

**Study**	***N***	**Menopause status**	**Hormonal augmentation**	**Daily dose (mg)**	**Treatment duration (weeks)**	**Results**
Kulkarni et al*.* (2001) [58]	36	Premenopausal	E (patch)	0.05	4	No differences in PANSS symptom scores between groups.
Kulkarni et al*.* (2001) [58]	24	Premenopausal	E (patch)	0.10	4	Reduced PANSS total, positive, negative, and general symptoms in treatment vs. control group.
Akhondzadeh et al*.* (2003) [59]	32	Premenopausal	EE	0.05	8	Reduced PANSS total, positive, and general symptoms in treatment vs. control group, no difference in PANSS negative symptoms.
Louzã et al*.* (2004) [50]	40	Unknown, age range 18–49	CEE	0.625	4	No differences in BPRS symptom scores between groups.
Kulkarni et al*.* (2008) [60]	102	Premenopausal	E (patch)	0.10	4	Reduced PANSS total positive and general symptoms in treatment vs. placebo group, no difference in PANSS negative symptoms.
Ghafari et al*.* (2013) [61]	32	Premenopausal	CEE	0.625	4	Reduced PANSS total, positive, negative, and general symptoms in treatment vs. placebo group.
Kulkarni et al*.* (2015) [62]	118	Premenopausal	E (patch)	0.10	8	Reduced PANSS total, positive, and general symptoms in treatment vs. placebo group, no difference in PANSS negative symptoms.
Kulkarni et al*.* (2015) [62]	124	Premenopausal	E (patch)	0.20	8	Reduced PANSS total, positive, and general symptoms in treatment vs. placebo group, no difference in PANSS negative symptoms.
Weiser et al*.* (2019) [63]	200	Premenopausal	E (patch)	0.20	8	Reduced PANSS total, positive, negative, and general symptoms in treatment vs. placebo group.

*PANSS* Positive and Negative Syndrome Scale, *BPRS* Brief Psychiatric Rating Scale, *E* estradiol, *CEE* conjugated equine estrogens, *EE* ethinyl estradiol, *EV* estradiol valerate

#### Raloxifene Augmentation

We found three meta-analyses that were published less than 5 years ago [51•, 52, 53]. Our search retrieved no original/novel RCT since these meta-analyses, apart from a subgroup analysis in a subsample of the RCT by Usall and colleagues [54, 55]. All original and relevant RCTs are summarized in Table 3.

**Table 3:** Randomized controlled trials investigating the effects of raloxifene augmentation therapy on symptoms in men and women with SSD

**Study**	***N***	**Sex**	**Menopause status**	**Daily dose (mg)**	**Treatment duration (weeks)**	**Results**
Kulkarni et al. (2010) [64]	22	F	Postmenopausal	60	12	No differences in PANSS symptom scores between groups.
Kulkarni et al. (2010) [64]	26	F	Postmenopausal	120	12	Reduced PANSS total and general symptoms in raloxifene vs. placebo group, no difference in PANSS positive and negative symptoms.
Usall et al. (2011) [65]	33	F	Postmenopausal	60	12	Reduced PANSS negative, positive, and general symptoms in raloxifene vs. placebo group.
Kianimehr et al. (2014) [66]	46	F	Postmenopausal	120	8	Reduced PANSS positive symptoms in raloxifene vs. placebo group, no difference in PANSS total, negative, and general symptoms.
Weickert et al. (2015) [67]	79	M, F	Unknown, age range 18–51	120	6	No differences in PANSS symptom scores between groups.
Kulkarni et al. (2016) [68]	56	F	Peri- and postmenopausal	120	12	Reduced PANSS total and general symptoms in raloxifene vs. placebo group, no difference in PANSS positive and negative symptoms.
Usall et al. (2016) [55]	70	F	Postmenopausal	60	24	Reduced PANSS total, negative, and general symptoms in raloxifene vs. placebo group, no difference in PANSS positive symptoms.
Weiser et al. (2017) [69]	200	F	Postmenopausal	120	16	Increased PANSS total, positive, negative, and general symptoms in raloxifene vs. placebo group.

*PANSS* Positive and Negative Syndrome Scale, *F* female, *M* male

The publication by De Boer and colleagues in 2018 was the most comprehensive meta-analysis, including nine studies in 561 patients with SSD, of which seven studies in postmenopausal women, one study in premenopausal women and men, and one study in men [51•]. Raloxifene was superior to placebo in improving total symptom severity (*N* = 482; Hedges’ *g* = 0.57, *p* = 0.009), as well as positive (*N* = 561; Hedges’ *g* = 0.32, *p* = 0.02), negative (*N* = 561; Hedges’ *g* = 0.40, *p* = 0.02), and general (*N* = 526; Hedges’ *g* = 0.46, *p* = 0.01) subscales, as measured by the PANSS.

The other meta-analyses specifically included RCTs in postmenopausal women, of which Wang et al. was the most comprehensive one [52, 53]. Corroborating the findings of de Boer and colleagues, Wang and colleagues found a superior effect of raloxifene as compared to placebo on PANSS total symptom severity, positive, negative, and general symptom severity (standard mean difference (SMD) − 0.22 to − 0.55, 95% confidence interval (CI) − 1.01 to − 0.02, *p* = 0.04–0.01; *I*^2^ = 74–79%). Overall, these studies indicate that adjunctive raloxifene improved PANSS positive, as well as total, negative, and general scores relative to placebo in women with SSD. As most studies have been conducted in postmenopausal women, there is most evidence for the beneficial effects of raloxifene for this group of women.

#### Safety and Tolerability of Hormonal Augmentation

Attitudes towards therapy with hormone-like agents tend to be negative due to the harmful effects that have been associated with its use in the past [56]. Although the prevalence of hormone replacement therapy for menopausal symptoms was high until the start of the current century, its use decreased due to two landmark studies who reported severe adverse effects. Current literature indicates that hormone replacement therapy is an acceptable and safe option for women before or within 10 years of menopause [57]. However, the safety of estrogen augmentation therapy in premenopausal women, and especially the safety of its long-term use (> 10 years) is still unknown.

Raloxifene has agonistic effects on the brain and bones, while having antagonistic effects on the breasts and uterus [28–30]. A recent review compared breast cancer risks in a sample of 116,317 raloxifene nonusers versus 1223 regular users of raloxifene and concluded that breast cancer-specific survival in raloxifene users was not worse than in nonusers, providing indirect evidence that raloxifene reduces breast cancer-related mortality [28]. In addition, a systematic review showed that the use of raloxifene is not associated with higher risks of endometrial cancer [30]. However, like all estrogens, raloxifene is associated with an increased risk of venous thromboembolic events (VTE). VTE risk seems highest at initiation and tends to decline with long-term use [30]. Overall, raloxifene can be considered more suitable for long-term use compared to estrogens.

#### Augmentation with Combined Contraceptives

We found no RCTs investigating the effects of combined contraceptives on symptom severity in women with SSD. Combined contraceptives (oral, patches, and vaginal ring) are widely prescribed to premenopausal women, including women with SSD. Despite the accumulating evidence of a beneficial effect of exogeneous estrogens on symptom severity in premenopausal women with SSD (Table 2), no studies investigated the effect of estrogenic contraceptives in premenopausal women with SSD. However, a recent meta-analysis evaluated psychotic exacerbations across the menstrual cycle in women with a psychotic disorder and showed that the rate of admissions during the perimenstrual phase was 1.48 times higher than expected (95% CI 1.31 to 1.67), indicating that hormonal fluctuations can negatively affect clinical outcome [22•]. By providing a regular daily dose of exogenous hormones, estrogenic contraceptives may have a stabilizing effect on endogenous sex hormone levels [27], especially when the stopping week is avoided. As estrogens are also known to affect the metabolism of several antipsychotics [17], the permanent use of a combined contraceptive may also help to stabilize the dose concentrations of antipsychotics. Instead, permanently lowering endogenous estrogen levels may also have a negative impact on symptom severity, considering the protective effects of estrogens. However, there are no studies that endorse any of these potential effects of combined contraceptives in women with SSD.

### Strategies to Reduce Antipsychotic-Induced Hyperprolactinemia

We found two large recent meta-analyses on prolactin-lowering strategies for antipsychotic-induced prolactin elevation [41•, 70•]. Zhang and colleagues [70•] specifically summarized studies on adjunctive therapies, while Lu and colleagues [41•] summarized both addition and switching strategies. Our search retrieved one additional clinical trial on adjunctive metformin (Fig. 4 in the supplement) [71].

#### Addition Strategies

The network meta-analysis of Zhang and colleagues [70•] included RCTs on adjunctive therapies with aripiprazole (*k* = 53), paeoniae–glycyrrhiza decoction (PGD) (*k* = 5), and metformin (*k* = 4), including 5550 patients (66% female). They composed nine different treatment groups based on treatment type and different dosing regimens. There were 23 RCTs that specifically included women, whereas 7 RCTs specifically included men. Aripiprazole of < 5 mg/day was found to be the most effective adjunctive therapy for reducing prolactin levels as compared to placebo and all other treatment groups, followed by aripiprazole at > 10 mg/day and aripiprazole 5–10 mg/day. Adjunctive PGD was associated with the least all-cause discontinuation rate, but since the quality standardization of PGD preparation is still lacking, this can currently not be considered as a treatment strategy and needs further research. Importantly, a subgroup analysis revealed a sex difference in the efficacy of the adjunctive treatments, showing a 25% smaller decrease in women as compared to men.

Lu and colleagues [41•] performed a series of meta-analyses including single-armed studies on adjunctive aripiprazole (*k* = 15), single-armed studies on adjunctive dopamine agonists (*k* = 9), RCTs on adjunctive aripiprazole (*k* = 12), and RCTs on adjunctive PGD (*k* = 3). All conducted meta-analyses contained high levels of heterogeneity. Based on their findings, it can also be concluded that adjunctive aripiprazole was the augmentation strategy with the best level of evidence, while the effect of PGD on prolactin levels was not significant.

Similar conclusions could be drawn from their network meta-analysis, which also included RCTs on adjunctive metformin (*k* = 2), adjunctive vitamin B6 (*k* = 1), adjunctive PDG (*k* = 5), and adjunctive dopamine agonists (*k* = 2). Although they found a significant effect of adjunctive vitamin B6, this was based on one study (*n* = 200) in which only males were included. In another network meta-analysis, they compared different dosing categories of adjunctive aripiprazole and concluded that aripiprazole doses of 5, 10, and > 10 mg/day were similarly effective, while prolactin elevation above 100 ng/ml was only significantly reduced at a dose of 5 mg/day. Aripiprazole at > 10 mg/day was associated with a higher incidence of side effects.

Our search retrieved one additional RCT on this topic. Zhu and colleagues [71] evaluated the effect of metformin addition on amisulpride-induced hyperprolactinemia (*n* = 86, 47% women). Prolactin levels in the metformin group were significantly lower compared to the placebo group after 8 weeks; this effect was observed in the whole group as well as in women separately.

#### Switching Strategies

Lu and colleagues evaluated the efficacy of switching strategies in a comprehensive meta-analysis of single-armed studies and a network meta-analysis on RCTs on switching strategies [41•]. The meta-analysis included 26 studies, of which 15 with aripiprazole, whereas other strategies included switching to quetiapine, clozapine, olanzapine, and brexpiprazole. The network meta-analysis included 6 RCTs on switching to aripiprazole, and one RCT on switching to quetiapine. Based on both analyses, switching to another antipsychotic appears to be an effective strategy to lower prolactin levels. When comparing different switching strategies, changing antipsychotic therapy to aripiprazole in titration and reducing the previous antipsychotic in titration has the best level of evidence, whereas switching to other antipsychotics (olanzapine or quetiapine) reached a low level of evidence.

## Discussion

This review provides an overview of evidence-based recommendations for pharmacotherapy for women with SSD, focusing on sex-specific dosing, hormonal augmentation, and correction of antipsychotic-induced prolactin elevation. Overall, we conclude that pharmacological treatment of women with SSD can be improved by (1) providing lower doses of most antipsychotics while accounting for hormonal life stages, (2) the implementation of augmentation strategies with estrogens and raloxifene, and (3) prolactin correction by the addition of, or switching to, aripiprazole.

Current literature consistently showed significantly increased dose-adjusted concentrations for most antipsychotic drugs in women as compared to men (10–70% higher for women), which suggests that sex-specific dosing is essential with a possible exception for quetiapine. Since estrogens interact with liver enzymes involved in metabolizing several antipsychotics, female-specific dosing may well be dependent on hormonal life stages (e.g., pre- or postmenopausal). Quetiapine does not show elevated serum concentrations in premenopausal women, which can be explained by the effects of estrogens in these women, resulting in a 20–30% higher activity of the enzyme CYP3A4 which is mostly responsible for the breakdown of these drugs [72, 73]. The same might be true for lurasidone, which has a pharmacokinetic profile similar to quetiapine [74], although this should be confirmed by TDM studies on sex differences. Similar research is required to determine sex differences in dose concentrations of other understudied antipsychotics (e.g., brexpiprazole, cariprazine).

The higher antipsychotic serum levels in women may be a potential reason of why women are more vulnerable to side effects of antipsychotics than men [18, 75•]. Currently, national guidelines and summaries of product characteristics do not consider female-specific dosing of antipsychotics, except for olanzapine, which mentions to possibility that women may need lower dose. Therapeutic drug monitoring (TDM) in female patients to prevent overmedication therefore seems appropriate, not only for clozapine, but also for olanzapine, amisulpride, aripiprazole, and potentially other antipsychotic drugs. TDM could especially be valuable during menopause, when strong hormonal fluctuations are expected to influence serum concentrations in an antipsychotic-specific manner.

Importantly, we found no studies on sex differences in dose concentrations of newer antipsychotics, such as lurasidone, cariprazine, and brexpiprazole. As lurasidone and cariprazine are primarily metabolized by CYP3A4 [74, 76], it could be expected that sex differences in serum concentrations for lurasidone are similar to those for quetiapine. For brexpiprazole, similar sex differences could be expected as for aripiprazole, as its metabolism is most similar to that of aripiprazole [74, 76]. However, these theoretical expectations warrant further research.

Augmentation with raloxifene was found to improve symptom severity especially in postmenopausal women. Conversely, in premenopausal women, the beneficial effects of estrogen augmentation were more consistent than those of raloxifene, but the latter can be used safely for a longer duration [30], which is not yet determined for estrogen augmentation. While the addition of raloxifene may be considered for premenopausal women, it cannot be recommended until its effectiveness is demonstrated in well-powered RCTs. Moreover, as both raloxifene and combined contraceptives increase the risk of VTE, raloxifene addition can only be considered in women that do not use this type of hormonal contraception. As contraceptive use may often be preferred in premenopausal women, another treatment strategy that could be investigated is the addition of raloxifene in combination with progesterone-only contraceptives. Combined contraceptives used continuously seems a reasonable choice from a theoretical perspective, given their potential to stabilize fluctuating estrogen levels. Yet, their effect in premenopausal women with SSD remains unknown. Although there have been several case-reports that endorse this theory, further research on potential benefits of contraceptives for premenopausal women with SSD is urgently needed.

Switching to a prolactin-sparing antipsychotic is effective in correcting prolactin to normal levels. However, this also carries the risk of precipitating a psychotic relapse, with a possible exception of clozapine which may even reduce relapse risk but may increase weight. Among the addition strategies, adjunctive aripiprazole is the best evidence-based option. Other evaluated adjunctive strategies (e.g., the addition of vitamin B6 or PGD) are less effective and/or less evidence based and may have a less favorable risk/benefit ratio. Aripiprazole addition also has potential disadvantages since polypharmacy is associated with increased side effects. However, low doses of aripiprazole (< 5 mg/day) appear to be most effective in correcting prolactin levels. Taken together, switching to aripiprazole or the addition of aripiprazole is the best evidenced strategies for prolactin correction. When choosing between the two, the risk of side effects must be weighed against the risk of relapse on an individual level.

We conclude that there is substantial evidence for three treatment strategies which can be applied to improve pharmacotherapy for women with SSD, i.e., a potential dose adjustment for antipsychotics other than quetiapine, considering hormonal augmentation therapy especially for the postmenopausal phase, and correction of increased prolactin levels. All three strategies can be implemented relatively simply, as they consist of affordable and available pharmaceutical interventions.

## Conclusions

**Fig. 1:**
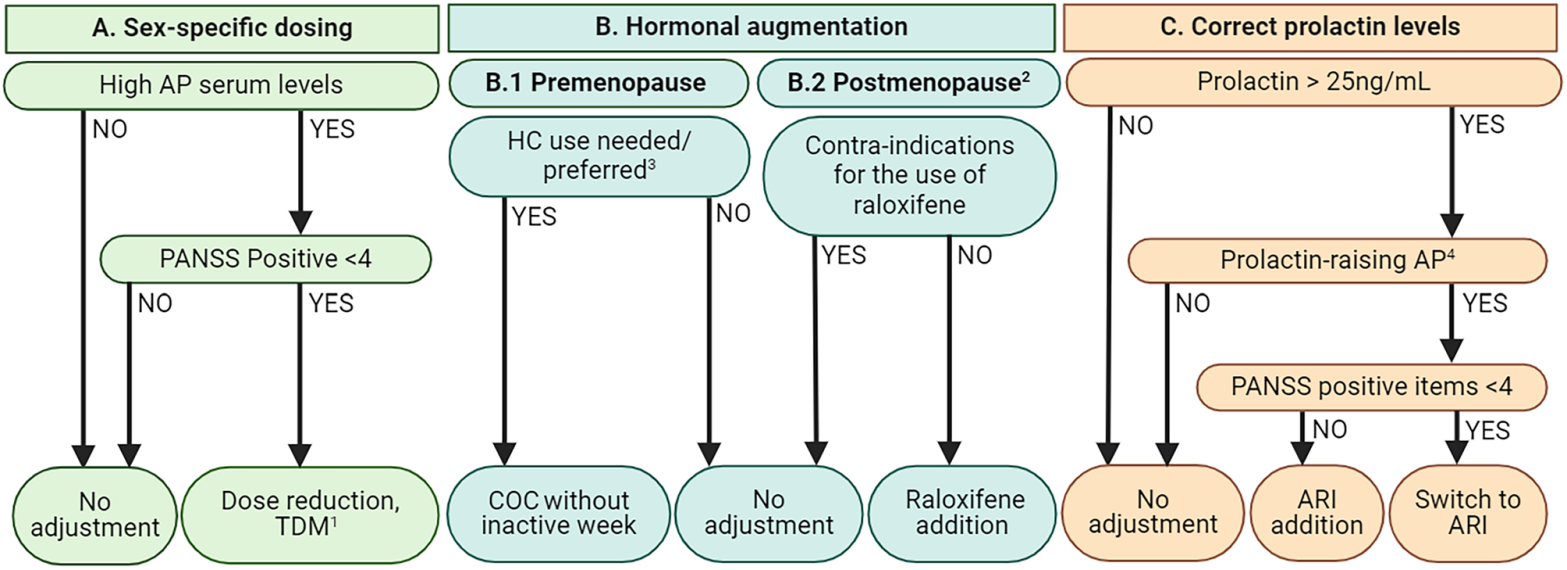
Evidence-based recommendations for female-specific pharmacotherapy, based on three pillars: sex-specific dosing, hormonal replacement, and correction of prolactin levels. Abbreviations: AP, antipsychotic; PANSS, Positive and Negative Syndrome Scale; TDM, therapeutic drug monitoring; COC, combined oral contraceptive; HC, hormonal contraceptive; ARI, aripiprazole. Footnotes: ^1^Based on Schoretsanitis et al. [77•]. ^2^Based on the Stages of Reproductive Aging Workshop (STRAW + 10). ^3^Should be based on shared decision, primarily led by preference and/or current use of the participant. ^4^Amisulpride, asenapine, chlorpromazine, haloperidol, lurasidone, olanzapine, paliperidone, and risperidone

Based current evidence, three recommendations for pharmacotherapy for women with SSD can be made. First, for women taking antipsychotics other than quetiapine with high or normal doses (conform SPC), a dose reduction might be advised. High blood levels (according to the therapeutic range established by Schoretsanitis [77•]) indicate that dose reduction and TDM is required. For quetiapine, menopausal status and age should be considered, as sex differences in dose-adjusted serum concentrations depend on estrogen levels and increase with age. Second, estrogen augmentation is effective for both pre- and post-menopausal women, but safety has not yet been studied for a longer duration than 6–8 weeks. Raloxifene augmentation can improve symptom severity during perimenopause and shortly after menopause without increasing the risk for breast and uterus cancer. However, at this point, we cannot provide any evidence-based recommendations for the use of estrogens, raloxifene, or combined contraceptives for premenopausal women, despite their potential value. Before any estrogen-like therapies are considered, women should always be screened for contra-indications including a (family) history of thrombo-embolism, which is a contra-indication. Third, antipsychotic-induced prolactin elevation can be reduced by switching to prolactin-sparing antipsychotics or (when relapse risk is significant), with the addition of aripiprazole. An overview of our recommendations, based on the currently available literature, is shown in Fig. 1. Eventually, these recommendations could be a first step towards female-specific pharmacotherapy. Although better outcome and fewer side effects for women with SSD are expected, the impact of these strategies needs to be confirmed in future RCTs.

### Supplementary Information

Below is the link to the electronic supplementary material.

Supplementary file1 (DOCX 67.3 KB)
