# Correction to: Evidence‑Based Recommendations for the Pharmacological Treatment of Women with Schizophrenia Spectrum Disorders

**DOI:** 10.1007/s11920-024-01520-5

**Published:** 2024-07-18

**Authors:** Bodyl A. Brand, Elske J. M. Willemse, Iris M. H. Hamers, Iris E. Sommer

**Affiliations:** Department of Biomedical Sciences and Systems, Cognitive Neurosciences, University of Groningen, University Medical Center Groningen (UMCG), Neuro Imaging Center 3111, Deusinglaan 2, Groningen, 9713 AW The Netherlands

**Correction to: Current Psychiatry Reports (2023) 25:723–733**.

10.1007/s11920-023-01460-6

The original version of this article unfortunately contained a mistake in the image of Fig. 1. Specifically, in section C. of this figure, the cut-off limit of prolactin is defined as 15 ng/L. The correct value of prolactin should be 25 ng/mL. The correct image is the following.

**Fig. 1:**
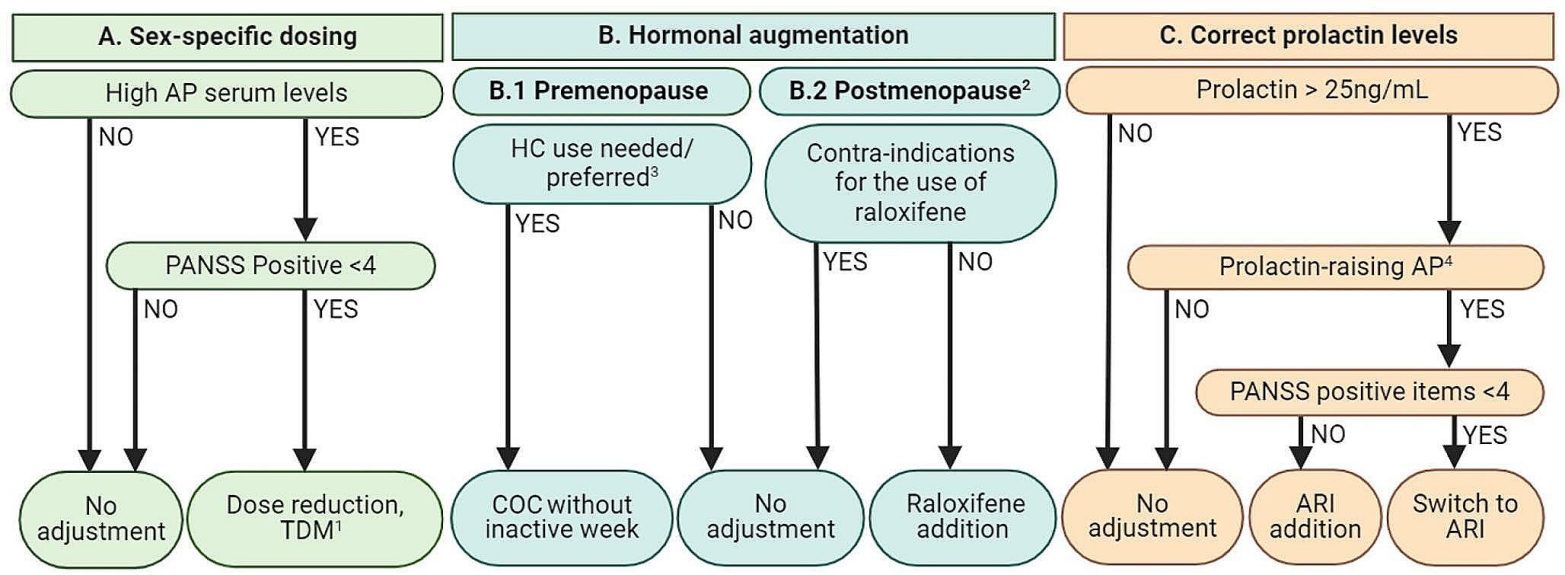
Evidence-based recommendations for female-specific pharmacotherapy, based on three pillars: sex-specific dosing, hormonal replacement, and correction of prolactin levels. Abbreviations: AP, antipsychotic; PANSS, Positive and Negative Syndrome Scale; TDM, therapeutic drug monitoring; COC, combined oral contraceptive; HC, hormonal contraceptive; ARI, aripiprazole. Footnotes: 1Based on Schoretsanitis et al. [77•]. 2Based on the Stages of Reproductive Aging Workshop (STRAW + 10). 3Should be based on shared decision, primarily led by preference and/or current use of the participant. 4Amisulpride, asenapine, chlorpromazine, haloperidol, lurasidone, olanzapine, paliperidone, and risperidone

The original version of this paper has been corrected.

