# Supplementary material for: Evidence-Based Recommendations for the Pharmacological Treatment of Women with Schizophrenia Spectrum Disorders
Source: Curr Psychiatry Rep. 2023 Oct 21;25(11):723–33. doi: 10.1007/s11920-023-01460-6 (PMC10654163; doi:10.1007/s11920-023-01460-6)
Supplement: Supplementary file 1 — Supplementary file1 (DOCX 67.3 KB) [file 11920_2023_1460_MOESM1_ESM.docx]

**Supplementary Information**

**Systematic search strategies**

**1** Dose-adjusted sex differences in antipsychotic serum concentrations

| **#1: Domain**  ((("serum concentration"[Title/Abstract] OR "serum concentrations"[Title/Abstract] OR "serum levels"[Title/Abstract] OR "serum level"[Title/Abstract] OR "blood level"[Title/Abstract] OR "dose concentration"[Title/Abstract] OR "dose concentrations"[Title/Abstract]) |
| --- |
| **#2: Determinant 1**  ("antipsychotic*"[Title/Abstract] OR "anti-psychotic"[Title/Abstract] OR "anti-psychotic"[Title/Abstract] OR "amisulprid*"[Title/Abstract] OR "aripiprazol*"[Title/Abstract] OR "chlorpromazin*"[Title/Abstract] OR "clozapin*"[Title/Abstract] OR "flupentixol*"[Title/Abstract] OR "haloperidol*"[Title/Abstract] OR "lurasidon*"[Title/Abstract] OR "olanzapin*"[Title/Abstract] OR "paliperidon*"[Title/Abstract] OR "quetiapin*"[Title/Abstract] OR "risperidon*"[Title/Abstract] OR "sulprid*"[Title/Abstract] OR "zuclopenthixol*"[Title/Abstract]) OR "antipsychotic agents"[MeSH Terms])) |
| **#3: Determinant 2**  (“femal*”[Title/Abstract] OR “sex”[Title/Abstract] OR “women”[Title/Abstract] |
| **#3: #1 AND #2** |

**2** Hormonal augmentation therapies

| **a. Estrogen** |
| --- |
| **#1: Domain**  ((((schizophren*[Title/Abstract] OR schizoaffective[Title/Abstract] OR psychosis[Title/Abstract] OR psychotic*[Title/Abstract] OR schizophreniform[Title/Abstract])) OR psychotic disorders[MeSH Terms])) |
| **#2: Determinant**  ((estrogen*[Title/Abstract] OR oestrogen*[Title/Abstract] OR estradiol[Title/Abstract])) |
| **#3: #1 AND #2** |

| **b. Raloxifene** |
| --- |
| **#1: Domain**  ((((schizophren*[Title/Abstract] OR schizoaffective[Title/Abstract] OR psychosis[Title/Abstract] OR psychotic*[Title/Abstract] OR schizophreniform[Title/Abstract])) OR psychotic disorders[MeSH Terms])) |
| **#2: Determinant**  ((((raloxifene[Title/Abstract] OR evista[Title/Abstract] OR selective estrogen receptor modulator*[Title/Abstract] OR SERM*[Title/Abstract])) OR raloxifene hydrochloride[MeSH Terms]) OR selective estrogen receptor modulators[MeSH Terms])) |
| **#3: #1 AND #2** |

| **c. Oral contraceptives** |
| --- |
| **#1: Domain**  ((((schizophren*[Title/Abstract] OR schizoaffective[Title/Abstract] OR psychosis[Title/Abstract] OR psychotic*[Title/Abstract] OR schizophreniform[Title/Abstract])) OR psychotic disorders[MeSH Terms])) |
| **#2: Determinant 1**  ((("oral contraceptives"[Title/Abstract] OR "oral contraception"[Title/Abstract] OR "contraceptive agent"[Title/Abstract] OR "birth control pill"[Title/Abstract] OR " combined contraceptives"[Title/Abstract]) OR oral contraceptives[MeSH Terms])) |
| **#3: #1 AND #2** |

**3** Strategies to reduce antipsychotic-induced hyperprolactinemia

| **#1: Domain**  ((((schizophren*[Title/Abstract] OR schizoaffective[Title/Abstract] OR psychosis[Title/Abstract] OR psychotic*[Title/Abstract] OR schizophreniform[Title/Abstract])) OR psychotic disorders[MeSH Terms])) |
| --- |
| **#2: Determinant 1**  ((prolactin*[Title/Abstract]) |
| **#3: Determinant 2**  (switch*[Title/Abstract] OR aripiprazol*[Title/Abstract] OR bromocriptin*[Title/Abstract] OR cabergolin*[Title/Abstract] OR “dopamine agonist” [Title/Abstract] OR metformin*[Title/Abstract])) |
| **#3: #1 AND #2 AND #3** |

**Supplementary Table** Inclusion criteria for each topic of interest

| 1. **Dose-adjusted sex differences in antipsychotic serum concentrations** |
| --- |
| 1. Population-based studies assessing sex differences in dose-adjusted concentrations of antipsychotics. |
| 1. **Hormonal augmentation therapies** |
| 1. Randomized, double-blind, placebo-controlled trials measuring the effect of estrogens, raloxifene or oral contraceptives on symptoms or cognitive functioning. 2. Study sample consisted of at least 30% female participants. |
| 1. **Strategies to reduce antipsychotic-induced hyperprolactinemia** |
| 1. Study sample consisted of at least 30% female participants. 2. Clinical trials 3. Published after November 1^st,^ 2021 (search cut-off date of Lu et al., 2022^35^) |

**Fig. 2** Prisma flow chart for sex-specific dosing studies

**252** Records identified through database searching

## **Screening**

## **Included**

## **Eligibility**

## **Identification**

**0** Additional records identified through other sources

**252** Records after duplicates removed

**252** Records screened

**245** Records excluded

**7** Full-text publications assessed for eligibility

**3** Full-text publications excluded
 **2** Not peer-reviewed journal
 **1** Meta-analysis

No RCT (n = 1)

**4** Publications included in qualitative synthesis

**Fig. 3** Prisma flow chart for estrogen augmentation therapy studies

**46** Records identified through database searching

## **Screening**

## **Included**

## **Eligibility**

## **Identification**

**0** Additional records identified through other sources

**46** Records after duplicates removed

**46** Records screened

**25** Records excluded

**3** No female patients

**18** No treatment with estrogens

**4** No SSD patients

**21** Full-text publications assessed for eligibility

**14** Full-text publications excluded

**9** Inappropriate outcome measure

**5** No original data

No original data (n = 3)

No RCT (n = 1)

**7** Publications included in qualitative synthesis

**Fig. 4** Prisma flow chart for strategies to reduce antipsychotic-induced hyperprolactinemia

**53** Records identified through database searching

## **Screening**

## **Included**

## **Eligibility**

## **Identification**

**0** Additional records identified through other sources

**53** Records after duplicates removed

**53** Records screened

**36** Records excluded

**11** Full-text publications assessed for eligibility

**10** Full-text publications excluded

**2** No female patients

**7** No original data

**1** No prolactin elevation at baseline

**1** Publication included in qualitative synthesis
